# *Brucella* dissociation is essential for macrophage egress and bacterial dissemination

**DOI:** 10.3389/fcimb.2014.00023

**Published:** 2014-03-05

**Authors:** Jianwu Pei, Melissa Kahl-McDonagh, Thomas A. Ficht

**Affiliations:** Department of Veterinary Pathobiology, Texas A&M University and Texas Agricultural Experiment StationCollege Station, TX, USA

**Keywords:** *Brucella* dissociation, macrophage cytotoxicity, egress and dissemination, plaque assay, infection foci

## Abstract

It has long been observed that smooth *Brucella* can dissociate into rough mutants that are cytotoxic to macrophages. However, the *in vivo* biological significance and/or mechanistic details of *Brucella* dissociation and cytotoxicity remain incomplete. In the current report, a plaque assay was developed using *Brucella* strains exhibiting varying degrees of cytotoxicity. Infected monolayers were observed daily using phase contrast microscopy for plaque formation while *Brucella* uptake and replication were monitored using an immunofluorescence assay (IFA). Visible plaques were detected at 4–5 days post infection (p.i.) with cytotoxic *Brucella* 16MΔ*manBA* at an MOI of 0.1. IFA staining demonstrated that the plaques consisted of macrophages with replicating *Brucella*. Visible plaques were not detected in monolayers infected with non-cytotoxic 16MΔ*manBA*Δ*virB2* at an MOI of 0.1. However, IFA staining did reveal small groups of macrophages (foci) with replicating *Brucella* in the monolayers infected with 16MΔ*manBA*Δ*virB2*. The size of the foci observed in macrophage monolayers infected with rough *Brucella* correlated directly with cytotoxicity measured in liquid culture, suggesting that cytotoxicity was essential for *Brucella* egress and dissemination. In monolayers infected with 16M, small and large foci were observed. Double antibody staining revealed spontaneous rough mutants within the large, but not the small foci in 16M infected monolayers. Furthermore, plaque formation was observed in the large foci derived from 16M infections. Finally, the addition of gentamicin to the culture medium inhibited plaque formation, suggesting that cell-to-cell spread occurred only following release of the organisms from the cells. Taken together, these results demonstrate that *Brucella*-induced cytotoxicity is critical for *Brucella* egress and dissemination.

## Introduction

*Brucella* is a genus of Gram-negative, facultative intracellular bacteria that cause brucellosis in a variety of animals and undulant fever in humans. Ten species have been described to date (Whatmore, 2009), and three species, *B. melitensis, B. abortus*, and *B. suis* provide the major threat to agriculture and public health worldwide (Boschiroli et al., 2001).

Macrophages/monocytes are the primary target cells in which *Brucella* replicate and cause persistent infection and as such are essential dissemination within the host (Baldwin and Winter, 1994; Liautard et al., 1996). Recent studies have shown that *Brucella* modulates the fate of infected macrophages and monocytes. Smooth *Brucella* infection inhibits macrophage and monocyte apoptosis by targeting the intrinsic (mitochondrial) and extrinsic (death receptor) pathways (Galdiero et al., 2000; Gross et al., 2000; Eskra et al., 2003; Fernandez-Prada et al., 2003; Tolomeo et al., 2003; He et al., 2006; Covert et al., 2009). In contrast, *Brucella* rough mutant infection results in type four-secretion system (T4SS) dependent macrophage cell death (Pei and Ficht, 2004; Pei et al., 2006, 2008b; De Jong et al., 2008; Zhong et al., 2009). It has been shown in a number of intracellular bacterial species that the regulation of host cell apoptosis is important to pathogenesis. Prevention of host cell apoptosis provides a hospitable intracellular niche for multiplication (Hacker and Fischer, 2002; Faherty and Maurelli, 2008) while induction of host cell death promotes bacterial release (Weinrauch and Zychlinsky, 1999; Gao and Kwaik, 2000b).

Although initial observations documenting rough *Brucella*-induced cell death in guinea pig macrophages occurred 50 years ago (Freeman et al., 1961), the mechanisms responsible have only been recently investigated (Pei and Ficht, 2004; Pei et al., 2006, 2008b; De Jong et al., 2008; Chen and He, 2009; Zhong et al., 2009; Chen et al., 2011), and the biological significance of the *Brucella* cytotoxicity remains undefined. In the current study, a plaque formation assay was developed to better evaluate *Brucella*-induced cytotoxicity. Comparison using *Brucella* strains with different levels of cytotoxicity provide direct evidence that cytotoxicity plays an important role in *Brucella* egress and dissemination in culture.

## Materials and methods

### Bacteria strains and media

Bacterial strains used in these experiments include *B. melitensis* 16M, rough mutants 16MΔ*manBA* and 16MΔ*manBA*Δ*virB2* (Pei et al., 2008b), and the rough *B. abortus* vaccine strain RB51. *Brucella* strains were routinely grown in tryptic soy agar (TSA) or tryptic soy broth (TSB) as described previously (Pei and Ficht, 2004).

### Cell culture and infection

Murine macrophage-like cells J774.A1 (ATCC, TIB-67) were grown in DMEM with 10% (v/v) fetal bovine serum, 1 mM L-glutamine, and 1 mM non-essential amino acid as described previously (Pei and Ficht, 2004). For plaque assays, 1.25 × 10^5^ cells were seeded into each well of a 24-well plate and incubated overnight at 37°C in atmosphere containing 5% CO_2_ prior to inoculation with *Brucella* at various multiplicities of infection (MOI). Infections were synchronized by centrifugation at 200× g for 5 min at room temperature and the plates were incubated at 37°C for 20 min. Cell monolayers were washed with PBS (pH 7.4) three times, and complete DMEM containing 50 μg/ml of gentamicin was added to kill extracellular bacteria with incubation at 37°C for 1 h (Pei and Ficht, 2004). *Brucella* uptake was determined following a 1-h incubation by washing the monolayers with PBS and lysing the cells with 0.5% (w/v) Tween 20 in distilled water. CFUs present in the lysates were determined as described previously (Pei and Ficht, 2004).

### Plaque formation assay (Oaks et al., 1985)

J774.A1 macrophages cultured in 24-well plates (1.25 × 10^5^ cells/well) were infected with *Brucella* as described above. Medium was replaced with 1 ml of warm (45°C) complete DMEM without gentamicin containing 1% (w/v) ultra-pure agarose (Gibco. Gaithersburg, MD). One milliliter of complete DMEM (with or without gentamicin) was added to each well following agarose solidification. Liquid media were changed every 2 days, and cell monolayers were observed daily using phase contrast light microscopy for plaque formation. Following incubation, liquid medium was removed and replaced with sufficient formalin to fix the cells and kill *Brucella* [3.7% (v/v) formaldehyde final] with incubation overnight at room temperature.

### Immunofluorescence assay (IFA)

Following fixation, the agarose was carefully removed and the cell monolayer washed with PBS. Infected cells were stained with goat anti *Brucella* serum and rabbit anti-rough *Brucella* monospecific serum (1:1000) in PBS-TT (PBS with 0.05% (v/v) Tween-20 and 0.05% (v/v) Triton X-100) for smooth and rough *Brucella*, respectively. Following three washes with PBS-T (PBS with 0.05% Tween-20), the cells were incubated with secondary antibodies including donkey anti goat IgG Alexa Fluor 488, chicken anti rabbit IgG Alexa Fluor 488, or chicken anti rabbit IgG Alexa Fluor 594 (Molecular Probes) (1:1000 in PBS-TT). Bacteria were revealed using IX70 fluorescence microscopy (Olympus).

### Double antibody staining

To differentiate rough mutants from smooth *Brucella*, J774.A1 macrophages were inoculated with 16M, 16MΔ*manBA*, or a mixture of 16M and 16MΔ*manBA* (10:1). The cells were fixed at 1 h after infection and incubated with rabbit anti *Brucella* mono-specific M serum (1:500 in PBS-TT) and goat anti *B. ovis* serum (1:500 in PBS-TT). Following three washes with PBS-T, the cells were incubated with chicken anti rabbit IgG Alexa Fluor 488 and donkey anti goat IgG Alexa Fluor 594 (1:1000 in PBS-TT). Bacteria were revealed using IX70 fluorescence microscopy.

### Plaque-forming unit

To enumerate plaque-forming units (PFUs) in each well, PFUs were averaged over five randomly selected fields in 16MΔ*manBA* infected monolayers using phase contrast and IX70 fluorescence microscopy (10× objective). Since, the area covered by each field under 10× objective lens is 1.798 mm^2^ and the total surface area is 200 mm^2^ the number of PFU/well is equal to the average PFU/field × (200/1.798).

## Results

### Plaque formation associated with *Brucella* cytotoxicity

**Figure 1:**
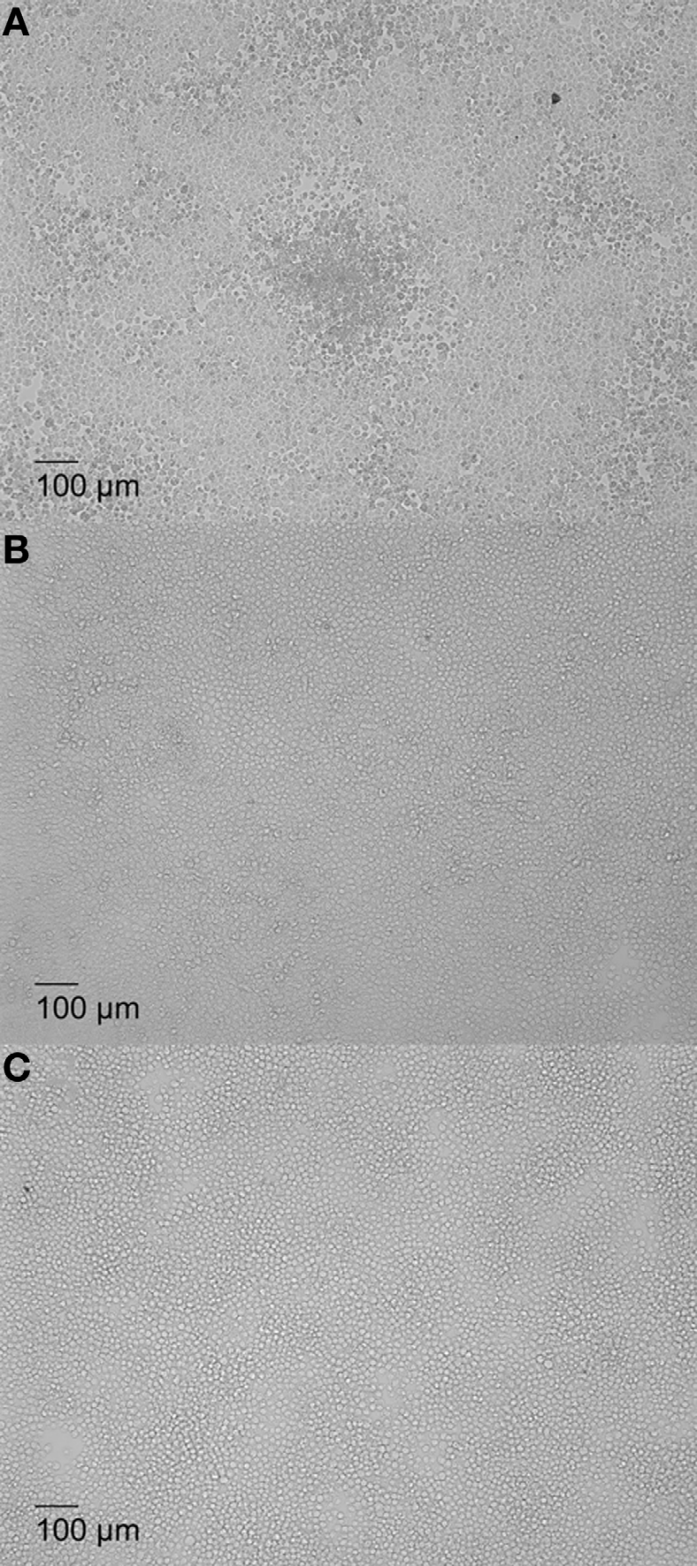
**Plaque formation in *Brucella*-infected macrophage monolayers associated with cytotoxicity**. J774.A1 macrophages were infected at an MOI of 0.1 with *B. melitensis* mutant 16MΔ*manBA* **(A)**, 16MΔ*manBA*Δ*virB2* 0.1 **(B)**, or left uninfected, as control **(C)**. The agarose (1% w/v) overlay and DMEM were added at 1 h p.i. as described in Materials and Methods, and incubated an additional 5 days. The cells were fixed overnight in 3.7% (w/v) formaldehyde and observed using IX70 microscopy. Scale bar, 100 μm.

Previous studies have shown that *Brucella* rough mutants proliferate in murine macrophage J774.A1 causing oncotic and necrotic cell death (Pei and Ficht, 2004; Pei et al., 2006). Therefore, infection with cytotoxic *Brucella* in macrophage monolayers with low MOI was predicted to produce plaques, resulting from replication and release of bacteria that infect neighboring cells to cause localized lysis. To test this hypothesis, a plaque assay was developed using the cytotoxic mutant 16MΔ*manBA* while the non-cytotoxic *Brucella* mutant 16MΔ*manBA*Δ*virB2* was employed as control (Pei et al., 2008b). In order to detect individual plaques, J774.A1 macrophages were infected with *Brucella* at low MOI (1.0 or 0.1). All the macrophages in the wells infected with 16MΔ*manBA* at 1 MOI were lysed within 4 days, and no individual plaques were observed. Plaques were visible between 4 and 5 days in monolayers infected with 16MΔ*manBA* at an MOI of 0.1 (Figure 1A). Neither cell death nor plaques were observed in cell monolayers infected with 16MΔ*manBA*Δ*virB2* at MOIs of 1 or 0.1 (data not shown) (Figure 1B). Since the MOI used was 0.1, at most only one in 10 macrophage are expected to be infected, and this was confirmed by IFA staining of infected cells at 1 h p.i. (shown in the following section). The results suggested that rough, cytotoxic *Brucella* replicated in macrophages causing cell death, and the bacteria released re-infected neighboring cells to cause a localized cell death. The process repeated itself until an area of dead cells (plaque) was observed (Figure 1A). MOI of 0.1 was used in all plaque assays described in this report unless otherwise specified.

**Figure 2:**
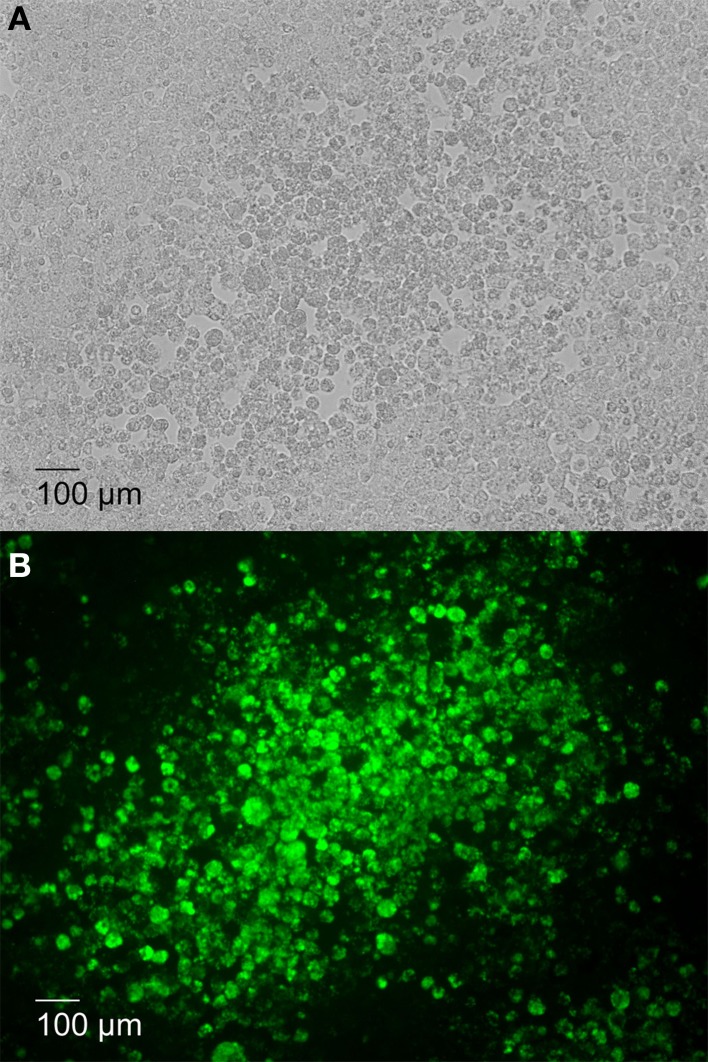
**Plaque formation corresponds with increased intracellular *Brucella* replication**. J774.A1 macrophages were infected with 16MΔ*manBA* at an MOI of 0.1. An agarose over lay and DMEM were added at 1 h p.i. and incubated an additional 4 days. Following fixation in 3.7% (w/v) formaldehyde, rough *Brucella* were visualized via IFA and IX70 microscopy under phase contrast field **(A)** and fluorescent field **(B)** (showing *Brucella* replication in green) microscopy. Scale bar, 100 μm.

To further determine whether the plaques observed in 16MΔ*manBA* infected cells (Figure 2A) were caused by *Brucella* replication, the infected cell monolayer was stained using the Immunofluorescence assay (IFA) for *Brucella* at 4 days p.i. The results revealed that significant replication of 16MΔ*manBA* occurred in cells comprising the plaques (Figure 2B). These results indicated that plaque formation was a direct result of the intracellular replication of the cytotoxic *Brucella* mutants.

### *Brucella* cytotoxicity was essential for bacteria egress and subsequent reinfection

**Figure 3:**
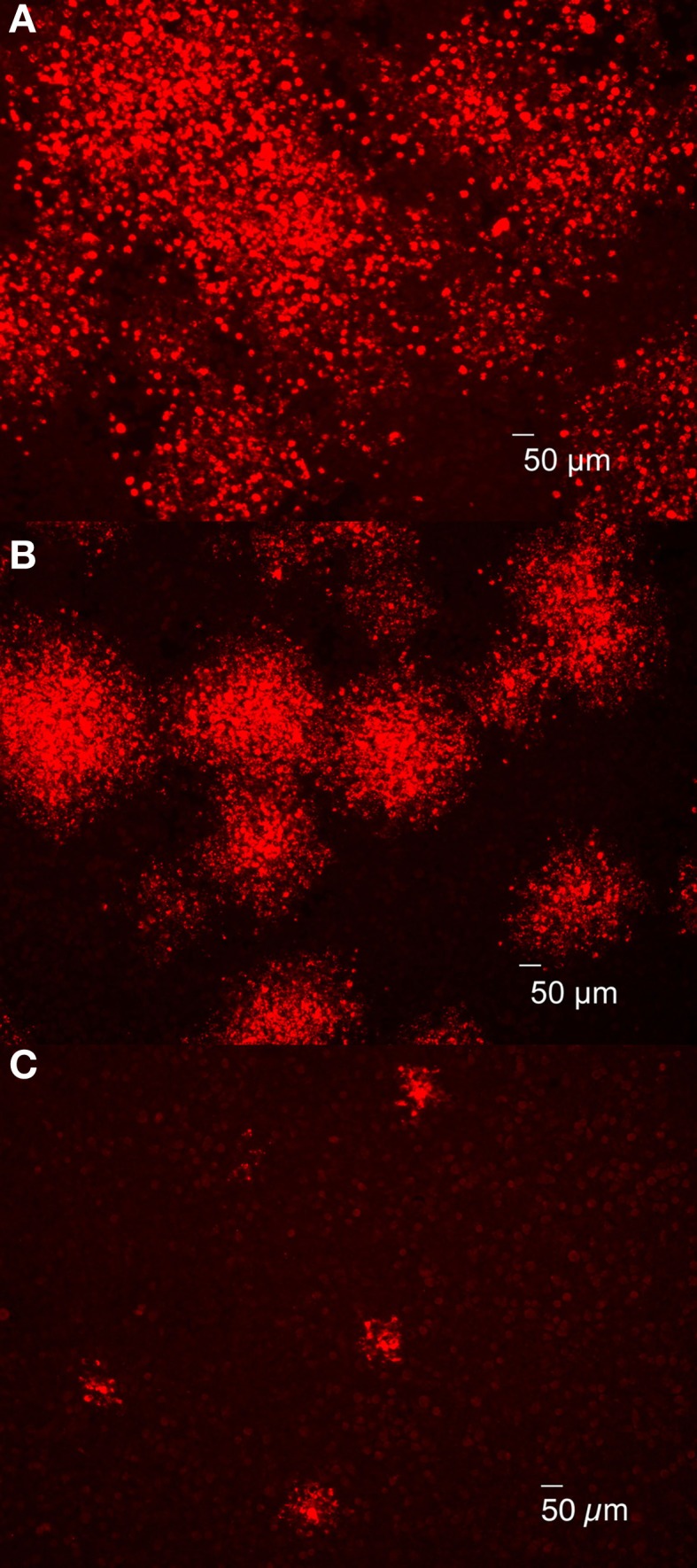
**Brucella cytotoxicity was essential for bacterial egress and subsequent re-infection**. J774.A1 macrophages were infected with 16MΔ*manBA* **(A)**, RB51 **(B)** or 16MΔ*manBA*Δ*virB2* **(C)** at an MOI of 0.1. Agarose overlay and DMEM were added at 1 h p.i. and incubated for 4 days. Following formaldehyde fixation, the cells were stained via IFA to detect rough *Brucella* observed using IX70 microscopy. Scale bar, 50 μm.

The results described in the previous section indicated that plaques were composed of dead, lysed or shedding cells containing replicating *Brucella*. However, differences in plaque size were apparent depending on the strain employed and it was hypothesized that plaque size was directly related to *Brucella* cytotoxicity, as measured by LDH release (Pei and Ficht, 2004). To test this hypothesis, J774.A1 macrophages were infected with *Brucella* strains exhibiting different levels of cytotoxicity: 16MΔ*manBA* (high cytotoxicity), 16MΔ*manBA*Δ*virB2* (no cytotoxicity), and *B. abortus* rough vaccine strain RB51 (low cytotoxicity) (Pei and Ficht, 2004; Pei et al., 2008b). *Brucella* replication and spread (designated foci) were detected by 4 days p.i. using IFA. As predicted, foci of infected macrophage monolayers containing RB51 were smaller than those in 16MΔ*manBA*-infected cells, but larger than the foci in monolayers infected with non-cytotoxic mutant 16MΔ*manBA*Δ*virB2* (Figure 3). These results suggested that cytotoxicity was directly related to *Brucella* dissemination and subsequent re-infection presumably as a result of enhanced egress, suggesting a possible role for cytotoxicity in the spread of infection.

**Figure 4:**
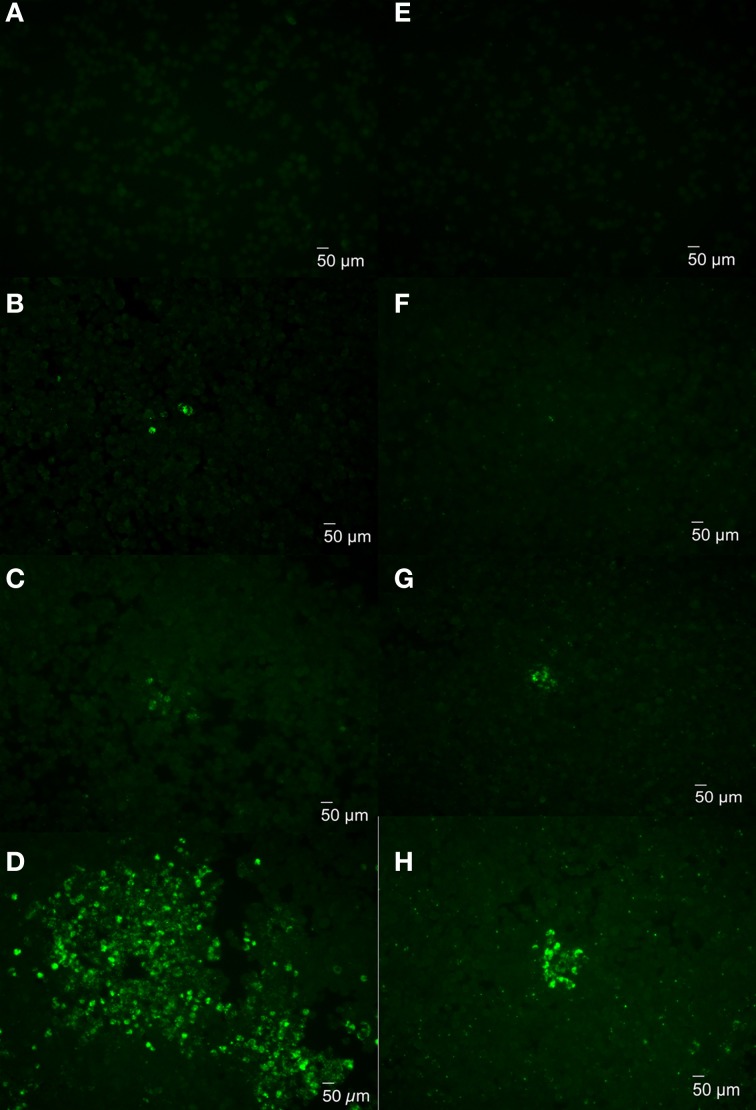
**Monitoring plaque formation during *Brucella* infection**. J774.A1 macrophages were infected with 16MΔ*manBA* **(A–D)** or 16MΔ*manBA*Δ*virB2* **(E–H)** at an MOI of 0.1. Agarose overlay and DMEM were added at 1 h p.i. and incubated an additional 4 days. The cells were fixed at 1 h **(A,E)**, 2 days **(B,F)**, 3 days **(C,G)**, and 4 days **(D,H)** p.i., and stained to visualize intracellular *Brucella* via IFA. Scale bar, 50 μm.

To further determine whether plaques can develop from an individual bacterium, uptake and replication of cytotoxic and non-cytotoxic *Brucella* were examined in J774.A1 monolayers infected with 16MΔ*manBA* or 16MΔ*manBA*Δ*virB2*. The infected monolayers were fixed at 1 h p.i. and visualized via rough-specific *Brucella* IFA staining to evaluate bacterial uptake. The cell monolayers were fixed at 2, 3, and 4 days p.i. and stained to monitor plaque (cytotoxicity) and focus (replication) formation. Staining of the infected cells following 1 h incubation confirmed a low-level of bacterial uptake, per infected cell (Figures 4A,E) and no demonstrable bacterial aggregation. By day 2, individual cells containing replicating *Brucella* were detected in the monolayers (Figures 4B,F), indicating that the bacteria replicated within the macrophages. By day 3, groups of cells containing *Brucella* were observed in the infected monolayers (Figures 4C,G). By day 4, large plaques consisting of dead cells were observed in the 16MΔ*manBA* infected cells using light microscopy (as shown in Figure 2A), and the cells within the foci were full of *Brucella* as revealed by IFA (Figure 4D). Although no plaques were observed in 16MΔ*manBA*Δ*virB2* infected monolayers at day 4 using light microscopy, foci containing large numbers of 16MΔ*manBA*Δ*virB2* were observed following IFA staining (Figure 4H). However, the foci detected by IFA were much smaller than those in the monolayers infected with 16MΔ*manBA* (Figure 4D). These results suggest that individual plaques or foci are formed by replication of a single bacterium, and further confirmed that the cytotoxicity was important for re-infection via extracellular spread to neighboring cells via egress from the host macrophage.

### *Brucella* dissociation assists organism dissemination

**Figure 5:**
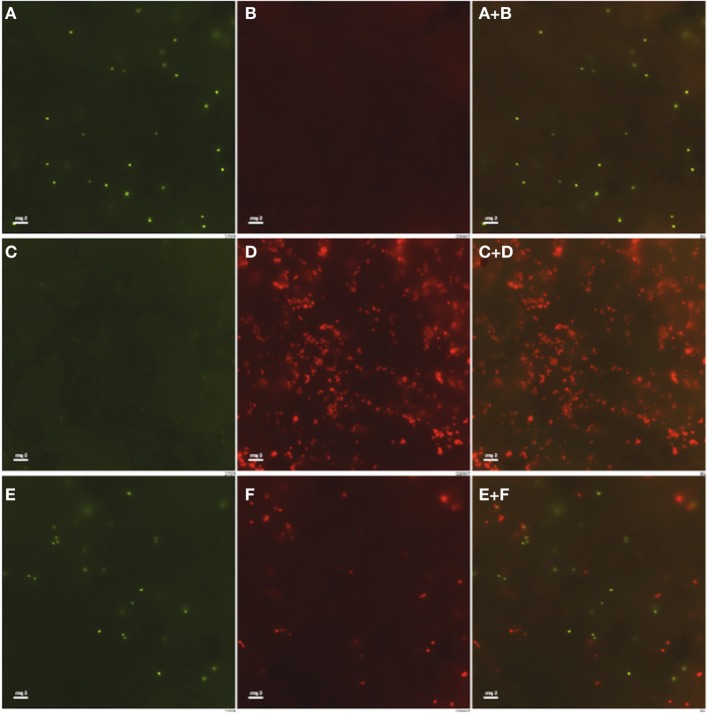
**Double antibody staining differentiates rough variants from smooth *Brucella***. J774.A1 macrophages were inoculated at an MOI of 50 with 16M **(A,B)**, 16MΔ*manBA* **(C,D)** or a 10:1 mixture of 16M and 16MΔ*manBA* **(E,F)**. The cells were fixed at 1 h p.i. and incubated with rabbit mono-specific anti-M serum (1:500 in PBS-TT) and goat anti-*B. ovis* serum (1:500 in PBS-TT). Intracellular smooth **(A,C,E)** and rough **(B,D,F)** *Brucella* were revealed following incubation with conjugated secondary antibodies chicken anti rabbit IgG Alexa Fluor 488 and donkey anti goat IgG Alexa Fluor 594 (1:1000 in PBS-TT). Panels **A+B, C+D, E+F** are derived by merging the appropriate channels. Scale bar, 5 μm.

Previous studies in our lab revealed that *Brucella* dissociation occurs *in vitro* and *in vivo* at elevated frequency and can result in an accumulation of rough variants in infected macrophages (Turse et al., 2011). However, previous studies have also revealed low levels of cytotoxicity associated with infection by wild type smooth *Brucella* at elevated MOI (Pei et al., 2008b). In order to determine whether ongoing dissociation, rather than low-level toxicity, is necessary for bacterial egress and dissemination, infection foci were evaluated for dissociation using a double antibody staining method described in the Materials and Methods. The results confirm the binding specificities of the rabbit anti-*Brucella* mono-specific M serum for wild type 16M (Figures 5A,C), and the goat anti-*B. ovis* serum for rough variants (Figures 5B,D). Antibody specificity and the capacity to distinguish smooth vs. rough *Brucella* during a mixed infection were confirmed using cells infected with a 10:1 mixture (smooth to rough) of *Brucella*, in which smooth *Brucella* were revealed in green (Figure 5E) and rough *Brucella* were revealed in red (Figure 5F).

**Figure 6:**
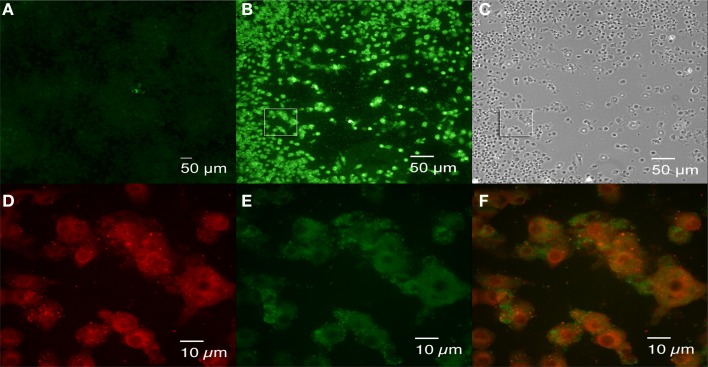
**Brucella dissociation detected in large foci following 16M infection**. J774.A1 macrophages were infected with 16M at an MOI of 0.1. Agarose overlay and fresh DMEM were added at 1 h p.i. and incubation was continued for an additional 4 days. Following fixation in 3.7 % (w/v) formaldehyde, double antibody staining (rabbit mono-specific anti-M serum and goat anti *B. ovis* serum) was performed as described in the legend to Figure 5. Small **(A)** and large **(B)** foci were observed. Cell death was observed in the large foci (panel **C**, a bright field image of panel **B**). Rough variants (red, **D**) arising during growth of the parental organism (green, **E**) were detected in the large foci. Panel **(F)** is the merged image of panels **(D,E)**, an enlarged square shown in **(B)**. Scale bars, 50 μm in **(A–C)**; 10 μm in **(D–F)**.

To detect rough dissociation in infected cells, J774.A1 macrophage monolayers were infected with 16M and fixed at 1 h and 4 days p.i. Because no antibiotic was added during experimentation, overgrowth of 16M in the media prevented further analysis beyond 4 days p.i. Double antibody staining of the cells fixed at 1 h p.i. confirmed a low-level bacterial uptake without bacterial aggregation or detectable dissociation (data not shown). Staining of the cells fixed at 4 days p.i. revealed two types of foci: small foci consisting of a few cells with replicating *Brucella* (Figure 6A) and large foci in which the cells that had not sloughed-off the surface of the plate were retained (Figures 6B,C). Double antibody staining confirmed the presence of significant numbers of rough *Brucell*a (Figures 6D,F) mixed with smooth *Brucella* (Figures 6E,F) in the large foci that were not detected in the small foci (data not shown). These results are consistent with the hypothesis that rough dissociation enhances *Brucella* dissemination.

To determine whether each *Brucella*-infected macrophage formed a plaque or focus of infection, PFUs in each well were evaluated via IFA staining at 4 days p.i. and compared with the uptake colony forming unit (CFUs) determined by gentamicin protection assay at 1 h p.i. The results indicated that 26.5% of the invading 16MΔ*manBA* formed plaques; only 4.6% of the invading 16M and 0.25% of the invading 16MΔ*manBA*Δ*viB2* formed foci.

### Inhibition of *Brucella* dissemination with the addition of gentamicin to growth medium

**Figure 7:**
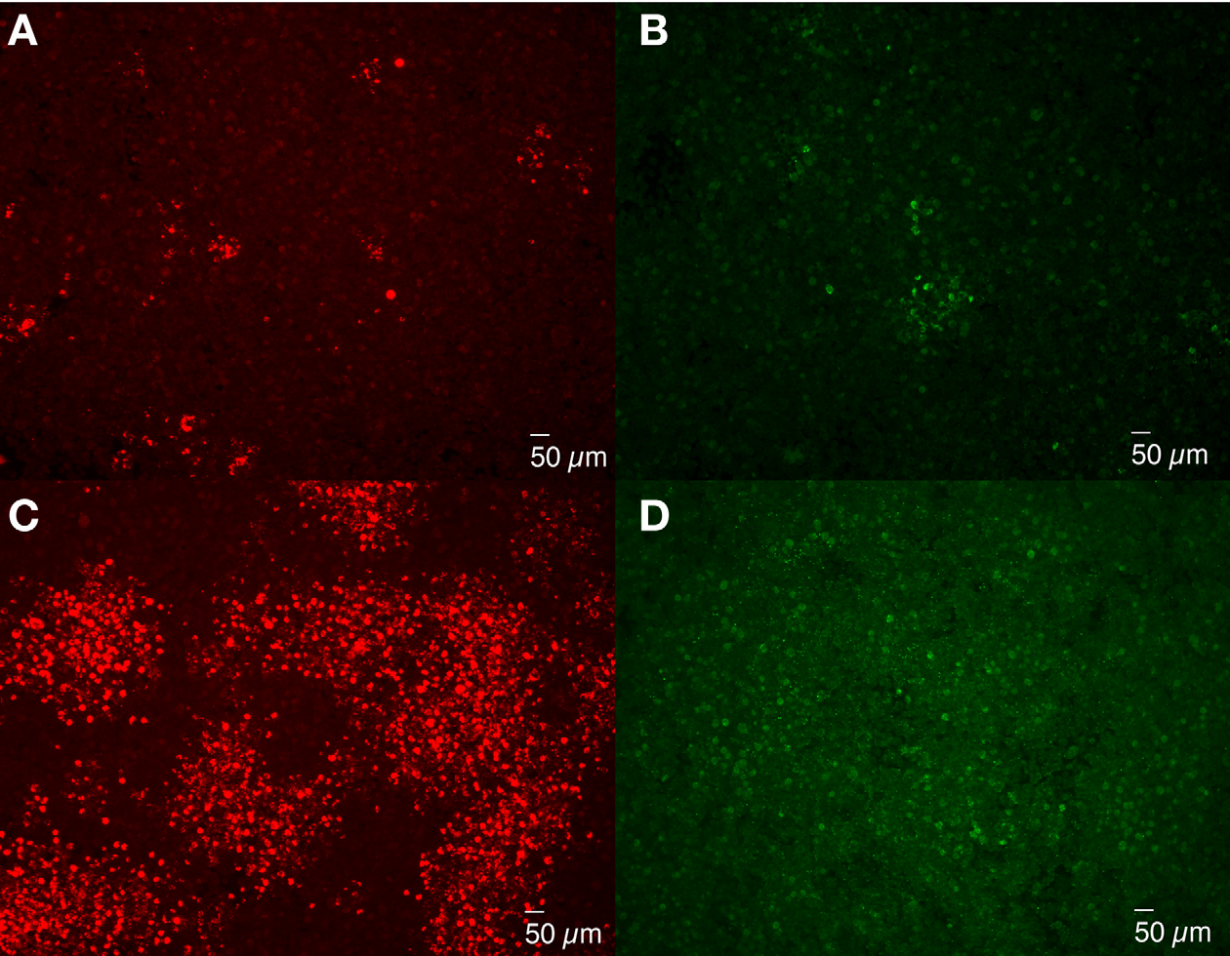
**Extracellular cell-to-cell dissemination of *Brucella***. J774.A1 macrophages were infected with 16MΔ*manBA* **(A,C)** or 16M **(B,D)** at an MOI of 0.1. An agarose overlay was added at 1 h p.i. along with fresh DMEM supplemented with **(A,B)** or without **(C,D)** 50 μg/ml of gentamicin. Following an additional 4 days of incubation, the cells were fixed and stained to visualize intracellular *Brucella* via IFA. Scale bar, 50 μm.

Bacterial spread from cell to cell can be accomplished by invasion of neighboring cells without release into the medium, such as *Listeria monocytogenes* (Mounier et al., 1990), or following release and re-infection of neighboring cells. To determine whether *Brucella* spread from cell to cell directly or via medium, experiments were performed in which gentamicin (50 μg/ml) was added to the overlay and DMEM so as to inactivate bacteria on the cell surface or released into the media and, as such, are not protected by intracellular uptake. Comparison of rough *B. melitensis* 16MΔ*manBA* and smooth 16M via IFA staining revealed small foci consisting of cells with replicating *Brucella* formed in monolayers infected with 16MΔ*manBA* (Figure 7A) and 16M (Figure 7B). But, neither plaques nor cell death were detected via phase contrast microscopy by 4 days p.i. (data not shown). In addition, the size of the foci was much smaller compared with those present in the infected monolayers without gentamicin treatment (Figures 7C,D). These results are consistent with the hypothesis that *Brucella* dissemination occurs via release into the medium with subsequent cell reinfection, and does not occur via cell-to-cell contact.

## Discussion

Pathogens have developed various strategies to evade host innate and adaptive immune systems (Finlay and McFadden, 2006), one of which is to manipulate host cell viability (Guiney, 2005). Similarly, both necrotic/apoptotic and anti-apoptotic cell death have been reported in macrophage during *Brucella* infection (Freeman et al., 1961; Gross et al., 2000; Fernandez-Prada et al., 2003; Pei and Ficht, 2004). Although several mechanisms have been thoroughly studied (Eskra et al., 2003; He et al., 2006; Pei et al., 2006, 2008b; De Jong et al., 2008; Chen and He, 2009; Zhong et al., 2009; Chen et al., 2011), their biological significance has not been established. The current study demonstrates that *Brucella* cytotoxicity enhances bacterial egress and dissemination.

An extensive amount of work has been performed regarding *Brucella* invasion and subversion of host cell functions thought to be critical to establishment of a hospitable replicative niche. However, another critical aspect of *Brucella* pathogenesis is organism egress from the host cell and continued dissemination. Seven potential exiting strategies or mechanisms used by different pathogens have been described in the literature to date (Hybiske and Stephens, 2008). *Legionella* generates pores in phagosomal or cellular membranes during different stages of infection inducing host cell cytotoxicity that is required for bacterial egress (Kirby et al., 1998; Alli et al., 2000; Gao and Kwaik, 2000a; Molmeret et al., 2002; Zink et al., 2002; Chen et al., 2004). *Salmonella* replication induces macrophage oncosis resulting in bacterial release from the cell (Sano et al., 2007). Similarly, our previous studies have revealed that rough *Brucella* infection induced pore formation on macrophage cell membranes to cause cell necrosis/oncosis (Pei et al., 2006). We provide support for this idea in the current study by demonstrating that *Brucella* cytotoxicity determines focus size, consistent with the idea that *Brucella* cytotoxicity is important for bacterial egress and dissemination.

Plaque formation was not apparent in the presence of gentamicin indicating that *Brucella* infection of neighboring cells occurs via extracellular dissemination of the organism. The reduced size of the foci formed under these conditions confirms the importance of extracellular spread of the organism to infection. Although the results obtained do not rule out the capacity of *Brucella* to disseminate using actin-based protrusion (used by *Listeria monocytogenes, Shigella flexneri, Rickettsia rickettsi, Rickettsia conorii, Burkholderia pseudomallei*, and *Mycobacterium marinum*), budding (by *Orientia tsutsugamushi*), or extrusion (*Chlamydia* spp.) (Hybiske and Stephens, 2008); the difference in the size of the foci observed suggests that *Brucella* dissemination is primarily the result of cell lysis and extracellular dissemination through the medium.

It has been proposed that two steps are required for *Legionella* release from infected cells. First, replicating *Legionella* form pores in the phagosomal membranes causing phagosome disruption. Second, bacteria released into the cytoplasm form pores in cell membrane resulting in cell lysis (Molmeret and Abu Kwaik, 2002). Our previous study has shown that rough *Brucella* are retained within intact vacuoles identified within dead macrophages (Pei et al., 2006), suggesting that phagosomal membranes are not disrupted, and suggesting that only a single step is involved in the release of *Brucella* from macrophages. A complete or well-defined description of *Brucella*-containing vacuoles (BCV) is not available, but the result described suggests that a putative *Brucella* “cytotoxin” might only form pores on the cell membrane, not on the BCV membrane. Inhibition of plaque formation by gentamicin treatment suggested that these BCVs do not protect the organisms from gentamicin. Since rough *Brucella* are sensitive to complement- and cationic peptide-mediated lysis, organisms released in intact BCV derived from lysed macrophages may be protected by the vacuoles and phagocytosed by surviving macrophages to start a new round of infection. Therefore, the one-step release may facilitate *Brucella* dissemination.

At the MOI used in the plaque assays, i.e., ≤0.1, bacterial uptake by infected cells is not expected to exceed more than one bacterium per infected cell. Yet, massive bacterial replication can be detected as soon as 4 days p.i. (Figures 2, 4). This is consistent with previous reports documenting rough *Brucella* mutant replication in HeLa cells and macrophages (Godfroid et al., 1998; Ugalde et al., 2000; Porte et al., 2003; Pei and Ficht, 2004; Pei et al., 2008b). These results demonstrate that *Brucella* cytotoxicity is not simply due to an uptake of excessively high numbers of *Brucella*, but derive from the replication of cytotoxic *Brucella* within the macrophages. The results also reveal that individual plaques are formed from cells infected with a single bacterium. The small foci associated with non-cytotoxic 16MΔ*manBA*Δ*virB2* result from their poor release from infected cells, despite significant levels of intracellular replication. It is not clear why the foci in non-cytotoxic strain infected monolayers was lower than that in cytotoxic strain infected monolayers. However, one possibility is that the foci formed by the non-cytotoxic strain are so small that they are easily missed during enumeration.

The T4SS is the predominant virulence factor identified in *Brucella* to date. It is widely accepted that the T4SS is essential for *Brucella* survival within host cells, and is consistent with the attenuated survival of *Brucella* Δ*virB* mutants in the mouse model (Hong et al., 2000). Yet, despite the well-defined role of the T4SS in *Brucella* intracellular trafficking (Comerci et al., 2001; Delrue et al., 2001; Celli et al., 2003), recent reports show that *virB* mutants survive in the host as well as wild type organisms for the first 3 days *in vivo* (Roux et al., 2007). Since the T4SS is essential for *Brucella* cytotoxicity (De Jong et al., 2008; Pei et al., 2008b; Zhong et al., 2009) and subsequent bacterial dissemination, *in vivo* attenuation of *virB* mutants could be explained by a failure to disseminate within the host just as well as failing to obtain a replication niche. This hypothesis is supported by our recent studies showing that the 16MΔ*manBA*Δ*virB2* mutant was cleared from infected mice within 1 week, while Δ*manBA* and Δ*virB2* single mutants persist in mice beyond 4 weeks (Pei and Ficht, unpublished data).

To evade the immune system for survival in the host, many pathogenic Gram-negative bacteria have the ability to alter their LPS structure, including smooth-rough variation (Lukacova et al., 2008). The current results suggest that the spontaneous appearance of rough variants from smooth *Brucella* may function in the dissemination of infection. *Brucella* dissociation, shown to be enhanced in acidic environments (Braun, 1946a), may induce dissociation and subsequently assist *Brucella* dissemination from within acidic phagosomes (Porte et al., 1999; Boschiroli et al., 2002). It should be pointed out that dissociated *Brucella* can still revert to smooth phenotype (Braun, 1947). Therefore, it is possible that some smooth *Brucella* dissociated into a rough phenotype when needed to disseminate, and can still revert to smooth phenotype following egress to resist intracellular killing.

It has been demonstrated that *Brucella* dissociation is genetically based (Braun, 1946a; Mancilla et al., 2010, 2012, 2013; Turse et al., 2011). Each individual bacterium may have a different dissociation rate. In the current study, the dissociation rate was not determined, but a study conducted by Braun showed that the percentage of rough organisms in individual cultures was different (Braun, 1946a). This could be the reason why different size foci were observed following smooth *Brucella* infection. Mancilla et al. demonstrated that phage integrase-mediated excision of genomic island 2 (GI-2) and ISBm1-mediated excision of *wbk*A glycosyltransferase gene were partially responsible for *Brucella* rough dissociation (Mancilla et al., 2010). Although knockout of these genes could not eliminate the dissociation, it would be interesting to determine the disseminating abilities of these mutants in comparison with the wild type. Starr et al. reported recently that forming autophagic *Brucella*-containing vacuoles (aBCV) promoted *Brucella* egress in HeLa cells. However, the phenomenon was not tested in macrophages in this report (Starr et al., 2012). Since autophagy is involved in *Brucella* survival and replication in macrophages (Qin et al., 2008; Guo et al., 2012; Starr et al., 2012), its role in *Brucella* egress from macrophage needs to be investigated.

**Figure 8:**
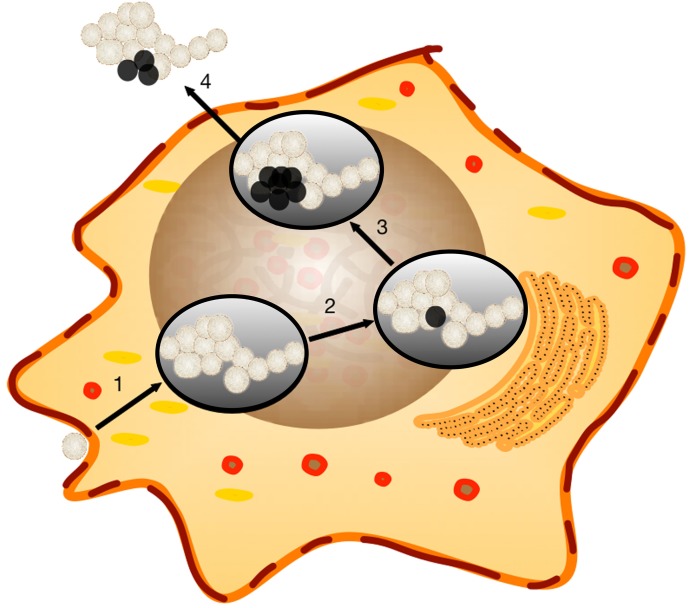
**Working model of *Brucella* host cell egress**. *Brucella* dissociation has been previously reported to continue unabated within the host cell. The properties of rough derivatives would lend themselves to bacterial dissemination and the induction of an inflammatory response sufficient to attract new target cells. Step 1, Smooth *Brucella*, that are resistant to extracellular killing mechanisms, successfully invades target macrophages where replication occurs within a *Brucella*-containing vacuole (BCV). Step 2, dissociation occurs in association with replication within the BCV as observed *in vitro*. Step 3, cytotoxic activity, enhanced in rough mutants, begins to break down the the cellular membrane. Step 4, *Brucella* are released from the cell and successive rounds of replication and dissociation continue. Rough *Brucella* infection and macrophage necrotic cell death induce inflammatory responses, which recruit more macrophages to the infection sites.

*Brucella* dissociation was observed more than 60 years ago (Stearns and Roepke, 1941; Braun, 1945, 1946a,b). However, the biological significance was not identified. Our current study revealed that *Brucella* dissociation enhanced bacterial dissemination, which may enhance *Brucella* virulence. A working model was proposed based on the results from this report and published studies (Figure 8). During smooth *Brucella* infection, the organism traffics to ER-like compartments and replicates (Celli et al., 2003). During replication, some of the organisms dissociate into a rough phenotype and accumulate in the host cells (Turse et al., 2011). The rough mutants with enhanced T4SS produce more cytotoxic factors (Pei et al., 2008b). Once rough mutant accumulation reaches a threshold level, the host cell will die from necrosis and apoptosis (Pei and Ficht, 2004; Pei et al., 2006; De Jong et al., 2008; Chen and He, 2009). The cell contents including the organisms will be released. Smooth *Brucella* will subsequently infect more macrophages and start a new round of replication and dissociation. Rough mutants may be killed by complement or other cationic peptide-mediated lysis (Allen et al., 1998). Rough *Brucella* induced cytokine and chemokine release (Rittig et al., 2003; Pei et al., 2008a) and macrophage necrotic cell death result in inflammatory responses, which in turn recruits more macrophages to the infection sites to help *Brucella* dissemination (Figure 8). This working model is strongly supported by the undulant fever presentation of human brucellosis.

### Conflict of interest statement

The authors declare that the research was conducted in the absence of any commercial or financial relationships that could be construed as a potential conflict of interest.
